# Metabolic subtype reveals potential therapeutic vulnerability in acute promyelocytic leukaemia

**DOI:** 10.1002/ctm2.964

**Published:** 2022-07-08

**Authors:** Ji‐Yong Sung, Woobin Yun, Hyun‐Young Kim, Hee‐Jin Kim, Jong Rak Choi, Sun‐Hee Kim, Chul Won Jung, Seung‐Tae Lee

**Affiliations:** ^1^ Department of Laboratory Medicine Yonsei University College of Medicine Seoul South Korea; ^2^ Brain Korea 21 PLUS Project for Medical Science Yonsei University Seoul South Korea; ^3^ Department of Laboratory Medicine and Genetics, Samsung Medical Center Sungkyunkwan University School of Medicine Seoul South Korea; ^4^ Dxome Co. Ltd. Seongnam‐si Gyeonggi‐do South Korea; ^5^ Department of Internal Medicine, Samsung Medical Center Sungkyunkwan University School of Medicine Seoul South Korea

**Keywords:** acute promyelocytic leukaemia, drug resistance, MAPK expression, metabolic reprogramming

Dear Editor,

Acute promyelocytic leukaemia (APL) is a rare blood cancer, classified as a subtype of acute myeloid leukaemia, and is treated with targeted therapy.^1^ Cancer metabolism has been studied extensively in solid cancers; however, metabolic reprogramming in blood cancer has not yet been studied extensively. We performed RNA‐sequencing analysis using bone marrow samples from 42 patients with APL as a discovery set. The hierarchical clustering and Pearson correlation similarity analyses identified two metabolic subtypes: low (metabolic subtype 1, MS1) and high (metabolic subtype 2, MS2) metabolic reprogramming (Figure 1A). All seven metabolic signatures^2^ in the discovery set showed significant differences except for integrated energy metabolism and lipids (*p* < .001) (Figure 1B). Public data from 323 patients with APL (GSE172057), used as a validation set, were concordant with those of the discovery set, with significant differences in 6 metabolic signatures, with no significant differences observed in energy metabolism (Figure 1C).

**FIGURE 1:**
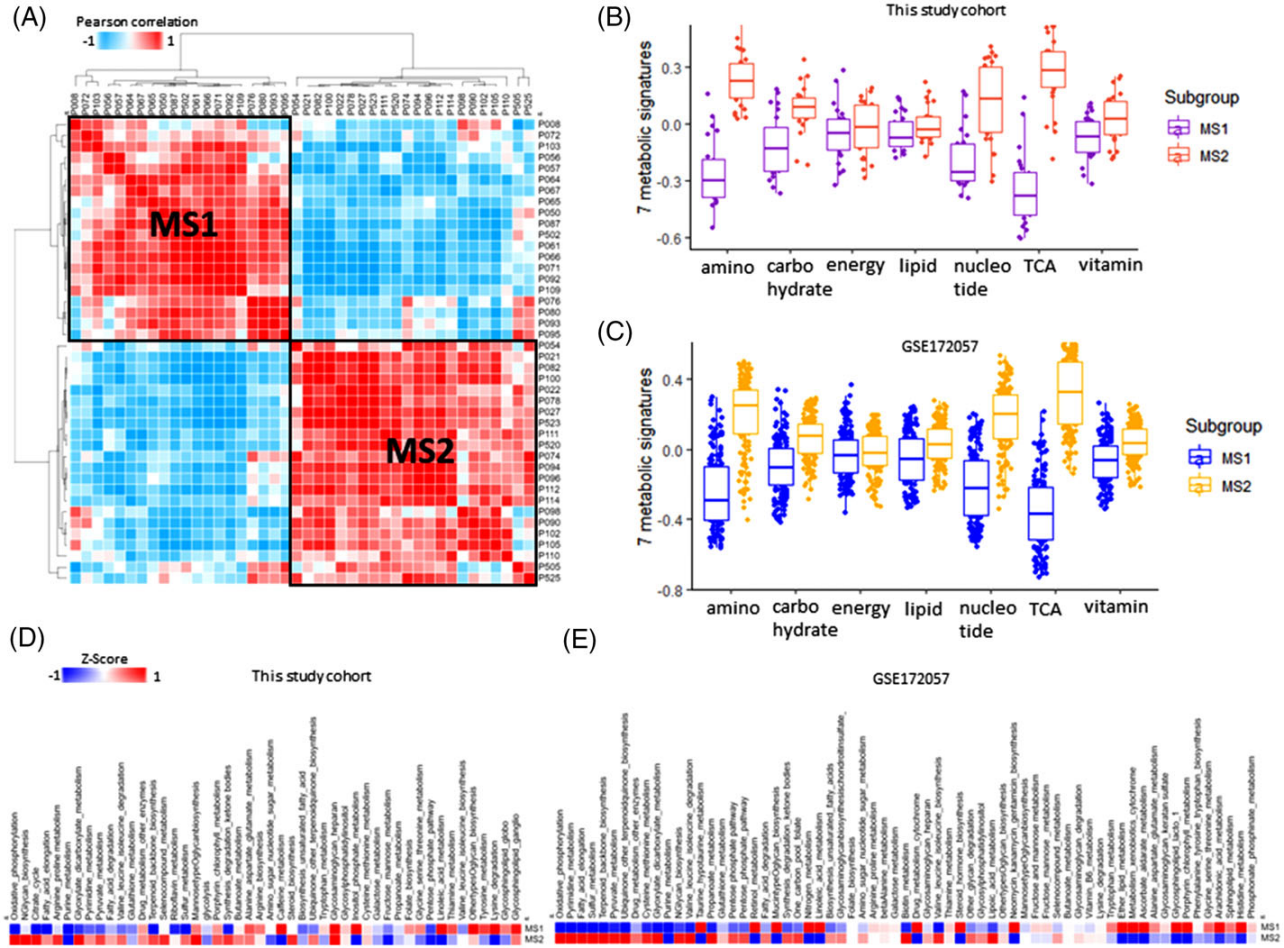
Metabolic landscape shows metabolic subtypes in acute promyelocytic leukaemia (APL). Statistical significance between the two groups (metabolic subtype 1 [MS1] and metabolic subtype 2 [MS2]) was *p* < .001: (A) Heat map of Pearson correlation similarity for seven metabolic signature scores (amino acid, carbohydrate, energy, lipid, nucleotide, tricarboxylic acid [TCA], and vitamin) shows two distinct metabolic subtypes (MS1 and MS2); (B) box plot of seven metabolic signatures between MS1 and MS2 in the discovery set. The gene enrichment score of the gene set variation analysis (GSVA) algorithm has a range of values between −1 and 1; (C) box plot of seven metabolic signatures between MS1 and MS2 in the GSE172057 validation set. The gene enrichment score of the GSVA algorithm has a range of values between −1 and 1; (D) heat map of 83 Kyoto encyclopaedia of genes and genomes (KEGG) metabolic pathways in MS1 and MS2 of the discovery set; (E) heat map of 83 KEGG metabolic pathways in MS1 and MS2 of the GSE172057 validation set

We performed an enrichment analysis of 84 Kyoto encyclopaedia of genes and genomes metabolic pathways and observed that the caffeine (*p* = .001) and inositol phosphate metabolism (*p* = .002) pathways were significantly upregulated in the MS1 subtype (Figure 1D). In contrast, most metabolic pathways were upregulated in the MS2 subtype, with 32 of the 52 metabolic pathways (63%) showing significant differences (Table S1). In the validation set, the oxidative phosphorylation pathway was highly expressed in MS2, whereas the taurine and hypotaurine metabolism pathway was upregulated in MS1 (Figure 1E) (Table S2). We compared 50 cancer hallmarks between MS1 and MS2 subtypes and observed significant differences in most of them (Figure 2A). Notably, retinoic acid (RA) and vitamin A signalling, which regulate hematopoietic stem cell (HSC) dormancy, were significantly upregulated in MS1 (Figure 2B). By contrast, genes upregulated by differentiation and proliferation renewal were highly expressed in MS2 (Figure 2B). Inhibition of RA signalling in HSCs maintains their primitive phenotype and promotes their self‐renewal.^3^ Genes involved in protein translation were more highly expressed in MS2 than in MS1, as evidenced by high telomere maintenance activity in MS2 (Figure 2C). We determined that ELF1, ZNF384, and KAT2A regulated the transcriptome in MS1 and TAF1, DLX1, and TP53 regulated the transcriptome in MS2 (Figure 2D), and that the autophagy signature was enriched in MS2 (*p* = .004) (Figure 2E). Among the three significant factors, KAT2A, ZNF384, and ELF1 were positively correlated with most RA signalling genes, and KAT2A was strongly positively correlated with NCOR2 and CREBBP (Figure 2F). Telomere maintenance mechanism (TMM) analysis revealed that the TERC–DKC1‐related pathway was significantly enriched in MS1 compared to MS2^4^ in terms of proliferation. RA signalling downregulates the TMM (Figure 2G) and telomere length through a pathway distinct from leukaemia cell differentiation.^5^ APL cells displayed low expression levels of autophagy genes and reduced autophagy activity,^6^ but autophagy activity was higher in MS2 than in MS1 (Figure 2E). High levels of RA inhibit autophagy; therefore, all‐trans RA (ATRA) and arsenic trioxide induce autophagy in APL, which provides a therapeutic advantage in patients with drug‐resistant APL. White blood cell and platelet count were lower in MS2 than in MS1 (*p* = .26 and *p* = .039; Figure 3A), which is known to be associated with an unfavourable prognosis.^7^

**FIGURE 2:**
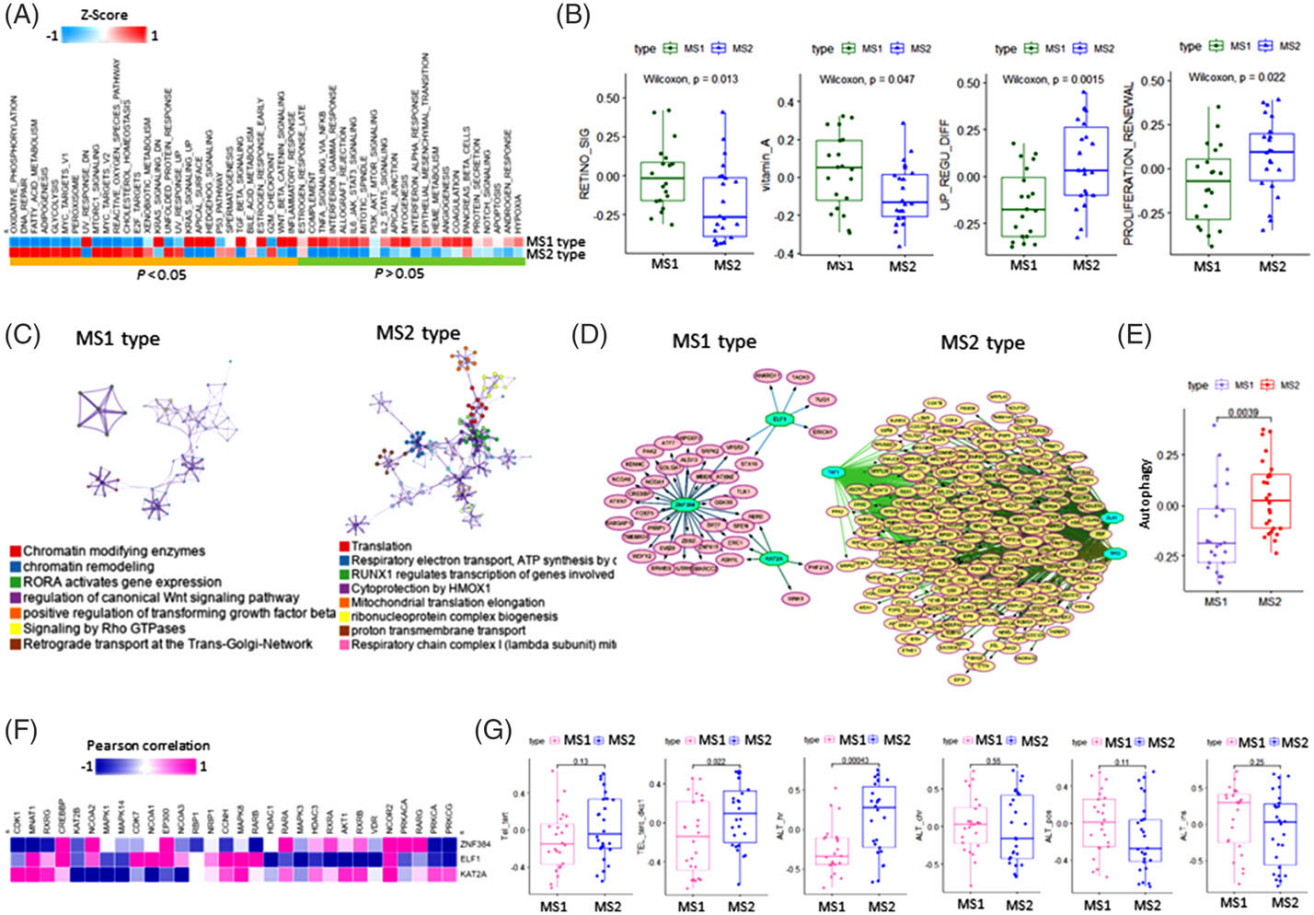
Metabolic subtypes show distinct cancer hallmarks: (A) Heat map of 50 cancer hallmarks in metabolic subtype 1 (MS1) and metabolic subtype 2 (MS2); (B) box plots of the retinoic signalling signature, vitamin A signature, and differentiation‐ and proliferation‐related genes in MS1 and MS2; (C) Gene Ontology (GO) network analysis of MS1 and MS2; (D) network analysis of transcription factor and target genes in MS1 and MS2; (E) box plots of the autophagy signatures of MS1 and MS2; (F) heat map of Pearson correlation between transcription factors (ZNF384, ELF1, and KAT2A) and retinoic acid signalling in MS1 and MS2; (G) box plots of the telomere maintenance mechanism in MS1 and MS2

**FIGURE 3:**
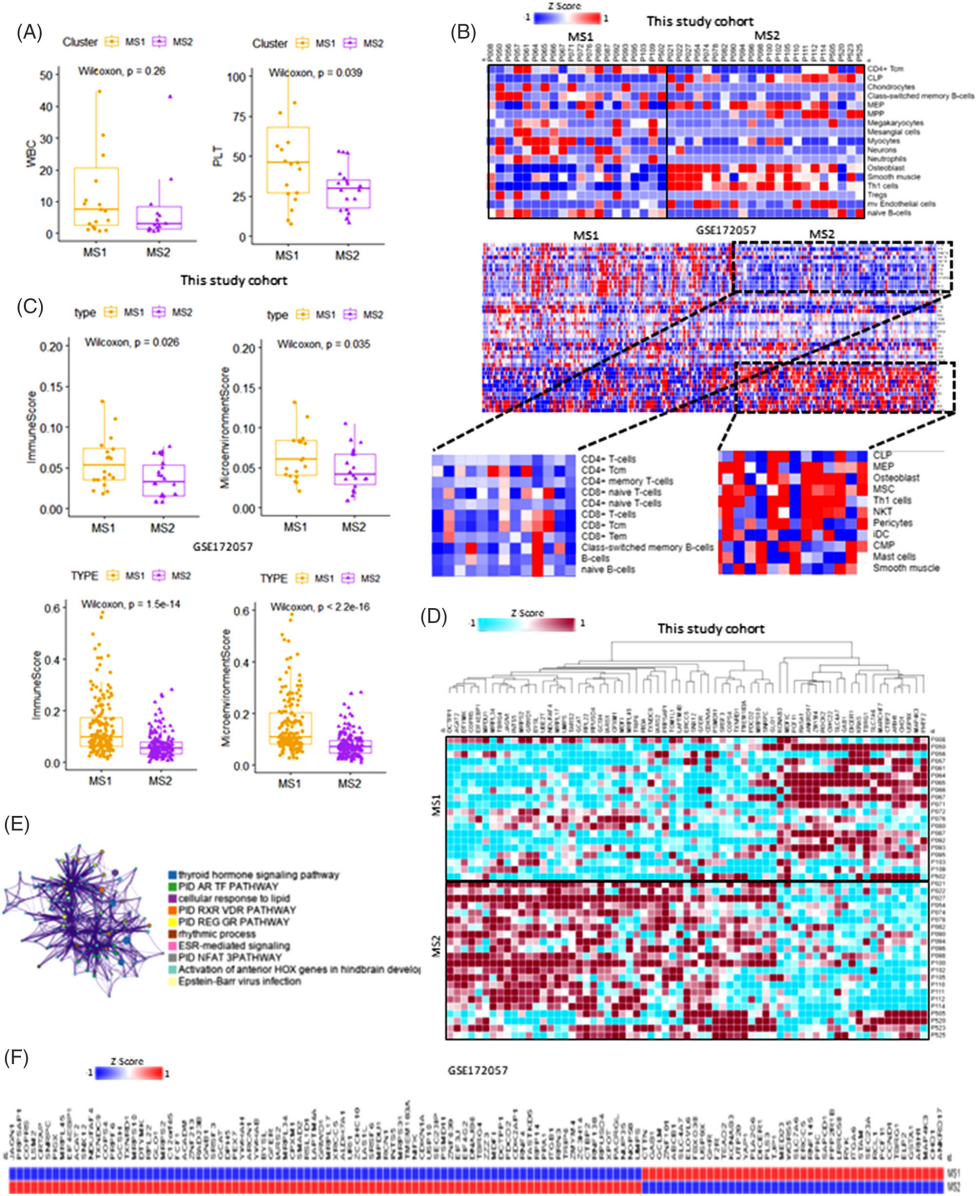
Metabolic subtypes show distinct transcriptional regulation: (A) box plots comparing clinical data of metabolic subset 1 (MS1) and metabolic subtype 2 (MS2); (B) heat map of differentially expressed immune cell signatures in the discovery set and the GSE172057 validation set; (C) box plots of the immune (a composite score of immune cell types) and the microenvironment scores (a composite score of immune cell types and stromal cell types) in the discovery set and the GSE172057 validation set; (D) heat map of differentially expressed haematopoietic stem cell signatures in the discovery set; (E) Gene Ontology (GO) analysis of retinoic acid signalling pathway genes; (F) heat map of differentially expressed hematopoietic stem cell signatures in the GSE172057 validation set

We confirmed that the levels of cells related to innate immunity, such as CD4^+^ central memory T cells and naïve B cells, were significantly higher in MS1 than in MS2 (Figure 3B). In the validation set, the immune landscape pattern was distinct. In the MS1 group, CD4, CD8, and B‐cell innate immune cell types were enriched, whereas in the MS2 group, CLP, MEP, mesenchymal stem cells, Th1 cells, and smooth muscle cells were enriched (Figure 3B). The immune and microenvironment scores in MS1 were significantly higher than those in MS2 (*p* = .026 and *p* = .035, respectively; Figure 3C). We performed a hierarchical cluster analysis of differentially expressed genes (DEGs) in the HSC signature. Most DEGs were upregulated in MS2, but *KCNAB3*, *MDF1C*, *PCF11*, and *RASA1* were higher in MS1 (Figure 3D). *KCNAB3*, *PCF11*, *ANKRD17*, and *TBRG1* were overexpressed in MS1 in both the discovery and the validation sets (Figure 3D,F). According to the Gene Ontology analysis, genes related to RA signalling were enriched in the thyroid hormone‐signalling pathway and lipid cellular response (Figure 3E). RA signalling affects the expression of other genes and induces cell differentiation. ATRA, which induces differentiation in APL, can cause drug resistance in some cases.^8^

Drug resistance can be predicted to be high in the MS2 subtype (Figure 4A). The predicted candidate drugs for MS2 included ATRA, VX‐702, sunitinib, and AZD7762. The target genes of these candidates included *MAPK11, MAPK12, MAPK14, PDGFR, KIT, VEGFR*, and *FLT3* (Figure 4B). We also confirmed that MAPK14 was highly correlated with sunitinib in the AML data set of genomics of drug sensitivity in cancer (Figure S1). For MS1, drugs such as SL‐0101‐1, CCT007093, and temsirolimus were predicted as candidates, with target genes such as *MTOR*, *POLA1*, *POLB*, *POLD1*, and *POLE* (Figure 4C). In the patient survival analysis, the death rate within 1 month was higher in MS2 (78%) than in MS1 (25%; *p* = .030) (Figure 4D). Most cases of early death were in the MS2 group, the causes were disseminated intravascular coagulation and intracranial haemorrhage, according to clinical data (Table S3). However, we confirmed that an excess number of oncogenic events occurred in the MS2 group, with high metabolic reprogramming and enhanced activity of drug‐resistance‐related genes.

**FIGURE 4:**
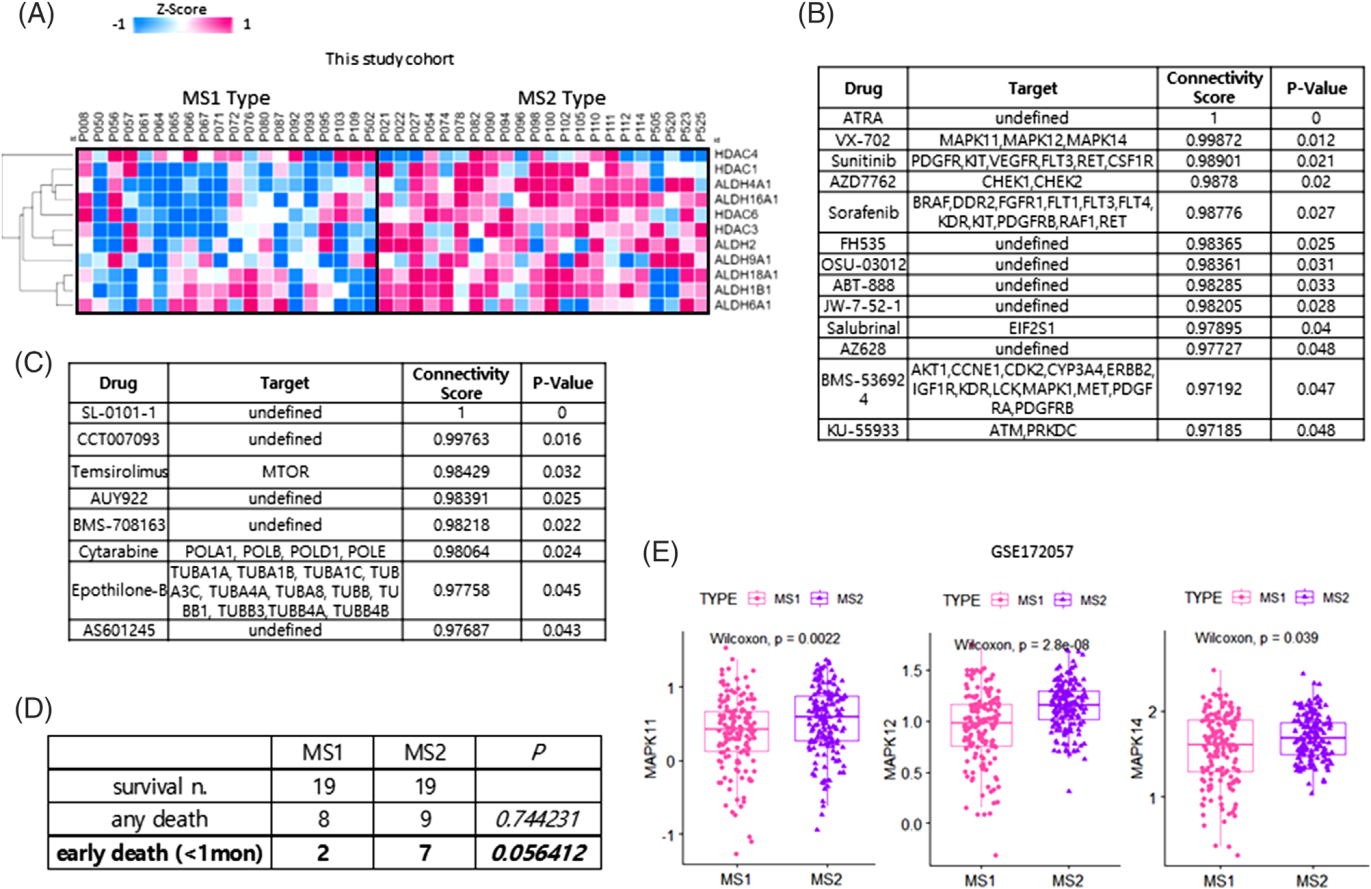
Therapeutic vulnerabilities of metabolic signature for acute promyelocytic leukaemia (APL) therapy: (A) heat map of the differential expression of genes related to drug resistance in APL between metabolic subset 1 (MS1) and metabolic subtype 2 (MS2) in the discovery set; (B) drug and target gene prediction using DeSigN (http://design.cancerresearch.my) for MS2; (C) drug and target gene prediction using DeSigN (http://design.cancerresearch.my) for MS1; (D) statistical survival prediction in the discovery cohort; (E) box plot of *MAPK11*, *MAPK12*, and *MAPK14* expression levels in MS1 and MS2

In the 323 samples from patients with APL in the validation set, *MAPK11*, *MAPK12*, and *MAPK14* were all significantly overexpressed in MS2 (Figure 4E). The increase of ATRA‐induced cell differentiation in APL NB4 cells requires inhibition of p38 MAPK phosphorylation.^9^ Through the stimulation of cell differentiation and accelerated cell migration to lymph nodes, RA could be essential in modulating the immune response to cancer and boosting antitumor immunity.^10^

## CONFLICT OF INTERESTS

The authors declare that there is no conflict of interest that could be perceived as prejudicing the impartiality of the research reported.

## Supporting information

Supporting Information

Figure S1 Predicted drug response from Genomics of Drug Sensitivity in Cancer (GDSC)

Table S1 KEGG metabolic pathway analysis in this study cohort
Table S2 KEGG metabolic pathway analysis in GSE172057
Table S3 Clinical data for APL patient
Table S4 mRNA sequencing statistics
