# Supplementary material for: Metabolic subtype reveals potential therapeutic vulnerability in acute promyelocytic leukaemia
Source: Clin Transl Med. 2022 Jul 8;12(7):e964. doi: 10.1002/ctm2.964 (PMC9270575; doi:10.1002/ctm2.964)
Supplement: Supplementary file 1 — Supporting Information [file CTM2-12-e964-s002.docx]

**Methods**

**Patient samples and RNA extraction**

We collected bone marrow samples from 50 patients at initial diagnosis with APL from a single center. The study was approved by the Institutional Review Board (IRB No. 2012-11-073-002). Participants provided written consent to participate in the study. Bone marrow cells were isolated and lysed, followed by total RNA extraction using TRIzol reagent (Life Technologies, CA, USA) according to the manufacturer’s instructions. All RNA samples were stored at −70 °C in RNAlater solution (Life Technologies).

**Library preparation and sequencing**

Ribosomal RNA was depleted using the Ribo-Zero Plus rRNA Depletion Kit (Illumina, CA, USA). Library preparation was performed using the TruSeq RNA Library Prep Kit v2 kit (Illumina) according to the manufacturer’s instructions. Sequencing was performed using the HiSeq 2000 system (Illumina) with paired-end chemistry (2 × 100 bp). Sequence quality parameters including total mean read, read length, uniquely mapped read, uniquely mapped read rate, and mapped length, are described in **Supplementary Table 4**.

**Sequencing read mapping and gene count reads**

Raw demultiplexed library reads were mapped to the human reference genome (*GRCh38*), and the reads were mapped using the STAR aligner tool 2.7.3a [1]. Counting reads per gene as a measure of gene expression was generated by the –*quantMode* GeneCount option of STAR and htseq-count, and all genes were annotated with *GRCh38* GTF format.

**Signature gene set analysis**

For metabolic subtype classification, the activities of the pathways for each sample were determined using RNA-seq expression profiles and single-sample gene set enrichment (ssGSEA) in the GSVA R package [2] and seven metabolic signatures (lipid, carbohydrate, energy, nucleotide, tricarboxylic acid [TCA] cycle, vitamin, and amino acid) [3]. We performed more than 1,000,000 runs to obtain statistical significance. We used 84 KEGG metabolic pathways for a more detailed analysis. Fifty cancer hallmark gene sets and gene sets related to retinoid signaling, vitamin A, upregulated differentiation, and proliferation renewal were downloaded from MSigDB (<https://www.gsea-msigdb.org/gsea/msigdb/>). For genes related to the telomere maintenance mechanism (TMM), a gene set from previous studies was used [4]. APL drug-resistance-related genes have been described previously [5].

**Drug prediction**

The DeSigN (<http://design.cancerresearch.my>) database and GDSC [6] were used for drug repositioning. The differentially expressed genes (DEGs) from the MS1 and MS2 groups were screened using the R package “limma,” [7] and genes with false discovery rate (FDR) < 0.01 were selected, and genes with positive and negative fold change values were used for distinguishing them. A functional protein association network was constructed for significant DEGs using STRING [8].

**Transcription factor and Gene Ontology [9] analysis**

Transcription factors were predicted using the Cytoscape plug-in iRegulon. The parameters for iRegulon were set at default (motif collection: 10 K (9713PWMs), track collection: 1120 Chip-seq tracks (ENCODE raw signals), TF prediction: maximum FDR on motif similarity 0.001). For GO analysis, only genes with FDR < 0.01 were selected among DEGs using METASCAPE [10].

1. Dobin, A., et al., *STAR: ultrafast universal RNA-seq aligner.* Bioinformatics, 2013. **29**(1): p. 15-21.

2. Hanzelmann, S., R. Castelo, and J. Guinney, *GSVA: gene set variation analysis for microarray and RNA-seq data.* BMC Bioinformatics, 2013. **14**: p. 7.

3. Peng, X., et al., *Molecular Characterization and Clinical Relevance of Metabolic Expression Subtypes in Human Cancers.* Cell Rep, 2018. **23**(1): p. 255-269 e4.

4. Sung, J.Y. and J.H. Cheong, *Pan-Cancer Analysis of Clinical Relevance via Telomere Maintenance Mechanism.* Int J Mol Sci, 2021. **22**(20).

5. Ozpolat, B., *Acute promyelocytic leukemia and differentiation therapy: molecular mechanisms of differentiation, retinoic acid resistance and novel treatments.* Turk J Haematol, 2009. **26**(2): p. 47-61.

6. Qin, Y., et al., *A tool for discovering drug sensitivity and gene expression associations in cancer cells.* PLoS One, 2017. **12**(4): p. e0176763.

7. Ritchie, M.E., et al., *limma powers differential expression analyses for RNA-sequencing and microarray studies.* Nucleic Acids Res, 2015. **43**(7): p. e47.

8. Szklarczyk, D., et al., *STRING v11: protein-protein association networks with increased coverage, supporting functional discovery in genome-wide experimental datasets.* Nucleic Acids Res, 2019. **47**(D1): p. D607-D613.

9. Pedro Domingos, M.P., *On the Optimality of the Simple Bayesian Classifier under Zero-One Loss.* Machine Learning, 1997. **29**(2-3): p. 103-130.

10. Zhou, Y., et al., *Metascape provides a biologist-oriented resource for the analysis of systems-level datasets.* Nat Commun, 2019. **10**(1): p. 1523.
