# Supplementary figures and images for: Metabolic subtype reveals potential therapeutic vulnerability in acute promyelocytic leukaemia
Source: Clin Transl Med. 2022 Jul 8;12(7):e964. doi: 10.1002/ctm2.964 (PMC9270575; doi:10.1002/ctm2.964)

## Slide 1
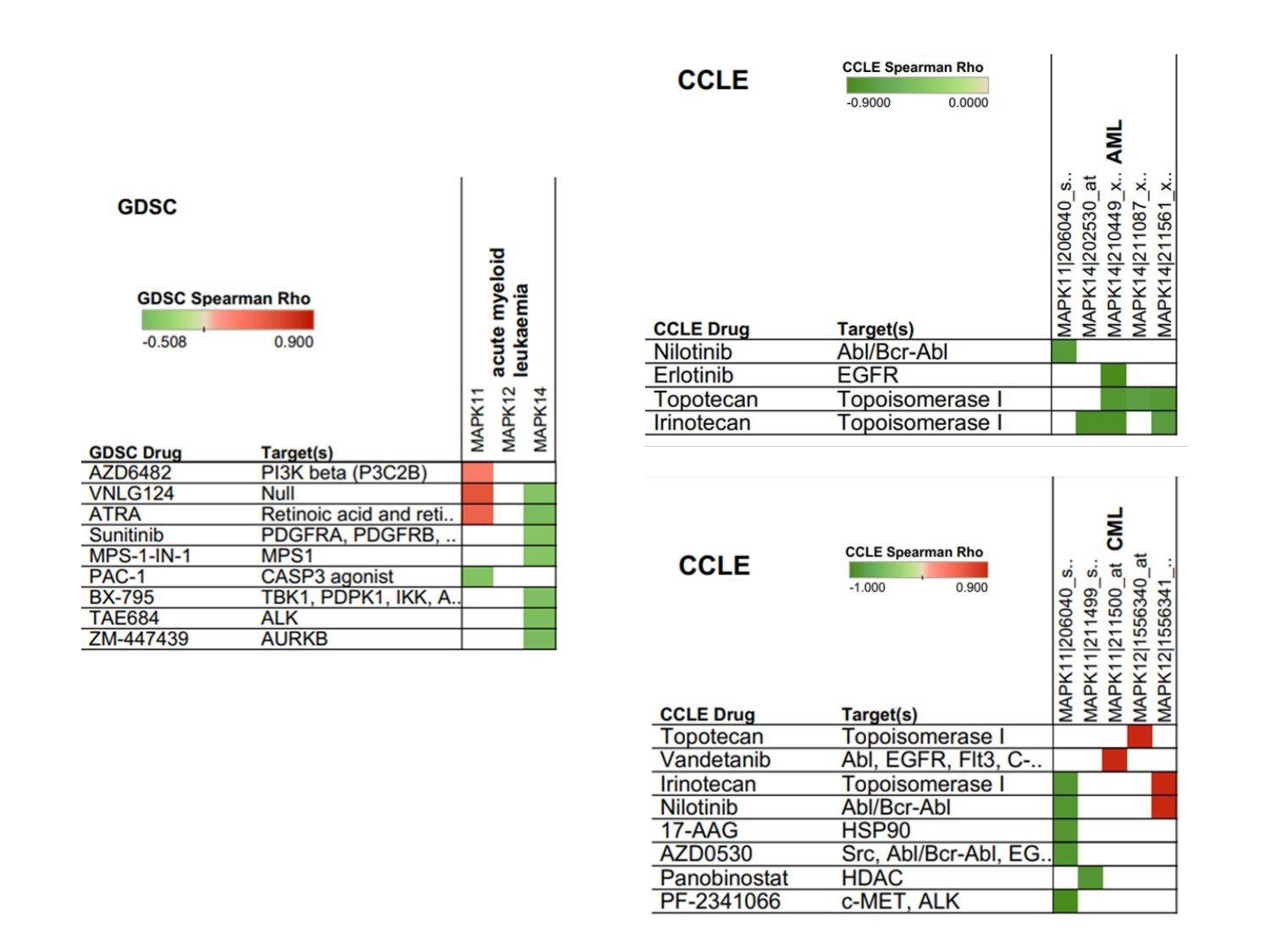

Supplement: Supplementary file 2 — Figure S1 Predicted drug response from Genomics of Drug Sensitivity in Cancer (GDSC) [file CTM2-12-e964-s003.pptx]
