# Supplementary material for: Metabolic subtype reveals potential therapeutic vulnerability in acute promyelocytic leukaemia
Source: Clin Transl Med. 2022 Jul 8;12(7):e964. doi: 10.1002/ctm2.964 (PMC9270575; doi:10.1002/ctm2.964)
Supplement: Supplementary file 3 — Table S1 KEGG metabolic pathway analysis in this study cohort Table S2 KEGG metabolic pathway analysis in GSE172057 Table S3 Clinical data for APL patient Table S4 mRNA sequencing statistics [file CTM2-12-e964-s001.pdf]

Supplementary Table 1. KEGG metabolic pathway analysis in this study cohort

ID	MS1	MS2	PVALUE
Oxidative_phosphorylation	-0.38943	0.28583	2.12E-11
NGlycan_biosynthesis	-0.16527	0.206356	1.33E-08
Citrate_cycle	-0.31683	0.173884	2.62E-07
Fatty_acid_elongation	-0.26573	0.117059	3.31E-07
Arginine_proline metabolism	-0.16918	0.153816	6.47E-07
Purine_metabolism	-0.43114	-0.35314	9.76E-07
Glyoxylate_dicarboxylate_metabolism	-0.23062	0.178206	1.5E-06
Pyrimidine_metabolism	-0.20955	0.116212	6.26E-06
Pyruvate_metabolism	-0.21731	0.145594	8.81E-06
Fatty_acid_degradation	-0.17942	0.10853	2.14E-05
Valine_leucine_isoleucine_degradation	-0.20505	0.130291	2.7E-05
Glutathione_metabolism	-0.19679	0.056343	3.74E-05
Drug_metabolism_other_enzymes	-0.11939	0.116409	4.32E-05
Terpenoid_backbone_biosynthesis	-0.26373	0.143366	4.35E-05
Selenocompound_metabolism	-0.15752	0.155011	5.95E-05
Riboflavin_metabolism	-0.24174	0.087299	6.23E-05
Sulfur_metabolism	-0.28818	0.128846	6.3E-05
MannosetypeOglycanbiosynthesis	-0.10795	0.202468	8.29E-05
glycolysis	-0.17163	0.037013	9.59E-05
Porphyrin_chlorophyll_metabolism	-0.11839	0.136153	0.000137
Synthesis_degradation_ketone bodies	-0.24912	0.15154	0.000139
Butanoate_metabolism	-0.14455	0.12183	0.000185
Alanine_aspartate_glutamate_metabolis	-0.09124	0.12471	0.000471
Arginine_biosynthesis	-0.10673	0.0898	0.000478
Amino_sugar_nucleotide_sugar_metabo	-0.15772	0.023401	0.000652
Caffeine_metabolism	0.251209	-0.12282	0.001058
Steroid_biosynthesis	-0.15685	0.156473	0.001188
Biosynthesis_unsaturated_fatty_acid	-0.20907	0.05228	0.001201
Ubiquinone_other_terpenoidquinone_bi	-0.20059	0.126269	0.001279
Tryptophan_metabolism	-0.13629	0.050394	0.001369
Glycosaminoglycan_heparan	-0.04971	0.212501	0.002045
Glycosylphosphatidylinositol	-0.13392	0.090153	0.002321
Inositol_phosphate_metabolism	0.018265	-0.14228	0.002362
Cysteine_methionine_metabolism	-0.14087	0.098497	0.00285
Galactose_metabolism	-0.17134	0.050925	0.003443
Fructose_mannose_metabolism	-0.19674	-0.00961	0.006444
Propanoate_metabolism	-0.16749	0.060039	0.007826
Folate_biosynthesis	-0.11634	0.072597	0.00809
Glycine_serine_threonine_metabolism	-0.11029	0.0365	0.008745
Pentose_phosphate_pathway	-0.22584	-0.00918	0.009753
Linoleic_acid_metabolism	0.107307	-0.03676	0.025424
Thiamine_metabolism	-0.08941	0.052421	0.028879
Valine_leucine_isoleucine_biosynthesis	-0.19487	0.079201	0.030173
OthertypesOglycan_biosynthesis	-0.04251	0.099497	0.034976
Tyrosine_metabolism	-0.0815	0.045626	0.036149
Lysine_degradation	0.025258	-0.08258	0.039853
Glycosphingolipid_globo	-0.1079	0.040495	0.044137
Glycosphingolipid_ganglio	-0.06002	0.123956	0.044658

Supplementary Table 2. KEGG metabolic pathway analysis in GSE172057

ID	MS1	MS2	PVALUE
Oxidative_phosphorylation	-0.35931	0.306098	4.59E-85
Pyrimidine_metabolism	-0.22582	0.193075	4.29E-48
Fatty_acid_elongation	-0.22057	0.150233	2.09E-32
Sulfur_metabolism	-0.27901	0.225187	4.56E-31
Terpenoid_backbone_biosynthesis	-0.25606	0.203066	1.64E-30
Pyruvate_metabolism	-0.18568	0.144251	5.68E-30
Ubiquinone_other_terpenoidquinone_biosynthes	-0.23316	0.186729	8.64E-30
Drug_metabolism_other_enzymes	-0.12974	0.09802	1.19E-29
Cysteine_methionine_metabolism	-0.1834	0.133258	3.8E-28
Glyoxylate_dicarboxylate_metabolism	-0.19795	0.172111	5.44E-27
Purine_metabolism	-0.44246	-0.37874	6.5E-27
NGlycan_biosynthesis	-0.16827	0.139311	1.56E-22
Valine_leucine_isoleucine_degradation	-0.18961	0.173264	2.02E-21
Taurine_hypotaurine_metabolism	0.10672	-0.1676	1.45E-20
Propanoate_metabolism	-0.18828	0.167103	7.5E-18
Glutathione_metabolism	-0.12282	0.071545	1.51E-17
Pentose phosphate pathway	-0.15491	0.114389	3E-17
Pentose_phosphate_pathway	-0.15491	0.114389	3E-17
Retinol_metabolism	0.08862	-0.09966	1.2E-16
Fatty_acid_degradation	-0.1314	0.096168	1.54E-15
MucintypeOglycan_biosynthesis	0.06513	-0.10949	5.63E-15
Synthesis_degradation_ketone bodies	-0.16125	0.111217	1.08E-14
One_carbon_pool_folate	-0.14908	0.120493	1.11E-14
Nitrogen_metabolism	0.093904	-0.12241	2.12E-14
Linoleic_acid_metabolism	0.092485	-0.1007	2.94E-14
Biosynthesis_unsaturated_fatty_acids	-0.15204	0.08736	6.36E-14
Glycosaminoglycanbiosynthesischondroitinsulfat	-0.13575	0.091519	2.87E-13
Folate_biosynthesis	-0.09836	0.049354	5.09E-12
Amino_sugar_nucleotide_sugar_metabolism	-0.11922	0.079768	1.64E-11
Arginine_proline metabolism	-0.08493	0.052815	5.07E-11
betaAlanine_metabolism	-0.08755	0.05778	5.49E-11
Galactose metabolism	-0.09158	0.047553	6.37E-11
Biotin_metabolism	-0.18397	0.172307	1.65E-10
Drug_metabolism_cytochrome	0.068908	-0.07349	4.39E-10
Glycosaminoglycan_heparan	-0.11464	0.077987	4.86E-10
Valine_leucine_isoleucine_biosynthesis	-0.19937	0.131107	9.6E-10
Thiamine_metabolism	-0.08232	0.059442	2.01E-09
Steroid_hormone_biosynthesis	0.066574	-0.06228	2.71E-09
Other_glycan_degradation	-0.14201	0.104525	2.87E-09
Glycosylphosphatidylinositol	-0.12765	0.08866	8.12E-09
Lipoic_acid_metabolism	-0.16881	0.151812	1.21E-08
OthertypesOglycan_biosynthesis	-0.11594	0.064697	4.96E-08
Neomycin_kanamycin_gentamicin_biosynthesis	0.078806	-0.14674	8.33E-08
MannosetypeOglycanbiosynthesis	-0.11663	0.047584	1.72E-07
Fructose and mannose metabolism	-0.0841	0.038914	1.94E-07
Fructose_mannose _metabolism	-0.0841	0.038914	1.94E-07
Selenocompound_metabolism	-0.12049	0.081455	3.11E-07
Butanoate_metabolism	-0.09635	0.053448	3.6E-07
Glycosaminoglycan_degradation	-0.10798	0.063733	3.74E-06
Vitamin_B6_metabolism	-0.11456	0.056702	8.81E-06
Lysine_degradation	-0.09499	0.055843	1.06E-05
Tryptophan_metabolism	-0.05693	0.027902	1.24E-05
Ether_lipid_metabolism	0.018991	-0.05418	3.95E-05
Metabolism_xenobiotics_cytochrome	0.038218	-0.04199	0.000148
Ascorbate_aldarate_metabolism	0.035143	-0.06162	0.000158
Alanine_aspartate_glutamate_metabolism	-0.05952	0.018958	0.000277
Glycosaminoglycan_keratan sulfate	-0.08582	0.021107	0.000336
Glycosphingolipid_lacto_1	0.002087	-0.05218	0.002697
Porphyrin_chlorophyll_metabolism	0.027554	-0.05609	0.002871
Phenylalanine_tyrosine_tryptophan_biosynthesis	-0.09415	0.019723	0.002988
Glycine_serine_threonine_metabolism	-0.04723	0.011413	0.005973
Arachidonic_acid_metabolism	0.021856	-0.02887	0.007083
Sphingolipid_metabolism	-0.0537	0.006304	0.008004
Histidine_metabolism	0.020472	-0.03799	0.008773
Phosphonate_phosphinate_metabolism	-0.08062	0.038147	0.009454

Supplementary Table 3. Clinical data in this study cohort (APL)

CaseN	SEX	Age	RNA-Seq	Hb-WBC-PLT-Reti	WBC	Hb	PLT	PT	PT.INR	APTT	Fibrinogen	FDP	D-dimer	high-risk	plt.score
P-057	F	69	O	9.0 g/dL - 16,570 /uL - 14K /uL	1.10	8.8	77	18.7	1.60	52.6	298	20	16.55	0	1
P-064	F	56	O	7.8 - 23060 - 35K - 1.04%	16.59	8.2	27	17.5	1.47	33.7	104	80	29.40	1	2
P-065	M	50	O	8.5 - 3690 - 35K - 2.89%	2.18	7.6	47	16.1	1.31	39.1	117		60.00	0	2
P-067	M	32	O	10.8 - 47290 - 41K - 1.31%	44.64	11.8	16	16.8	1.38	35.0	172		60.00	1	2
P-072	F	48	O	6.4 - 29330 - 62K - 1.43%	30.93	7.9	84	18.6	1.57	43.1	138		55.42	1	1
P-076	M	57	O	5.6 - 9340 - 56K - 1.60%	9.34	5.6	56	15.1	1.19	35.0	313		60.00	0	1
P-080	M	18	O	9.6 - 5380 - 31K - 1.44%	7.15	9.9	27	15.4	1.21	36.7	110		60.00	0	2
P-092	F	57	O	10.3 - 2100 - 61K-2.1%	4.21	8.5	42	16.5	1.34	35.9	170	16	34.90	0	2
P-093	M	23	O	9.8 - 2890 - 54K - 1.78%	2.89	9.8	54	15.7	1.27	38.4	90		16.97	0	1
P-095	M	31	O	9.1 - 560 - 55K - 2.77%	0.86	10.5	46	16.0	1.30	42.3	80	76	27.99	0	2
P-109	M	54	O	8.5 - 5080 - 91K - 2.70%	1.48	10.0	114	15.5	1.25	38.8	262		54.98	0	0
P-008	M	26	O	6.4-173400-58K-0.62	157.60	8.2	32	16.2	1.41	33.4	201	160	29.00	1	2
P-050	M	60	O	8.0 - 4.53 - 70K - 0.58%	3.66	5.7	10	15.9	1.32	30.7	240	80	35.00	0	2
P-056	M	73	O	7.7 - 23.92 - 64K	24.74	6.5	23	19.3	16.30	47.3	333	80	134.00	1	2
P-061	M	72	O	6.4 - 1,040 - 36K - 1.67%	7.51	8.7	174	14.0	1.07	38.7	241		31.13	0	0
P-066	F	92	O	9.8 - 700 - 49K - 1.80%	0.66	9.1	45	15.6	1.26	35.0	286	160	60.00	0	2
P-071	F	68	O	8.6 - 1680 - 8K - 1.78%	10.40	6.9	132	14.0	1.11	35.5				1	0
P-087	M	63	O	8.5 - 11320 - 47K - 0.71%	9.18	10.3	8	19.1	1.63	43.7	86			0	2
P-103	M	45	O	9.9 - 46190 - 78K - 2.91%	51.44	9.6	59	23.2	2.16	47.5	100	205	60.00	1	1
P-502	F	46	O												
P-027	M	58	O	7.6 - 8260 - 33K - 1.1%	9.02	8.5	16	17.1	1.29	38.4	44	80	36.00	0	2
P-074	M	64	O	7.1 - 2270 - 31K -2.12%	1.70	9.2	16	16.0	1.27	32.6	90		43.03	0	2
P-090	F	50	O	9.4 - 3070 - 56K - 0.76%	3.03	7.9	34	16.7	1.39	36.0	222	160	70.00	0	2
P-094	M	37	O	8.6 - 3880 - 46K - 0.87%	2.50	9.7	36	15.6	1.26	36.3	132	69	21.51	0	2
P-096	M	25	O	6.6 - 3980 - 40K - 1.23%	4.10	6.2	11	17.8	1.49	36.8	139	197	60.00	0	2
P-102	M	26	O	7.6 - 20340 - 41K - 2.81%	5.43	10.0	8	15.9	1.29	37.6	105	59	18.95	0	2
P-110	M	62	O	10.2 - 750 - 20K - 1.69%	1.18	11.2	39	15.7	1.27	38.9	141	155	33.45	0	2
P-111	M	64	O	7.9 - 4490 - 59K	0.59	7.9	52	20.2	1.78	49.8	48		60.00	0	1
P-112	M	28	O	6.8 - 830 - 40K - 1.23%	0.74	7.2	52	15.3	1.23	40.8	251		6.44	0	1
P-114	F	49	O	8.9 - 0790 - 28K	0.72	6.7	33	16.1	1.29	33.3	130		6.04	0	2
P-021	M	68	O	4.4-75000-18K	68.05	14.8	33	17.4	1.34	35.9	100			1	2
P-022	M	69	O	9.8-3080-20K-1%	3.04	10.7	23	25.4	1.97	40.0	28	40	35.30	0	2
P-054	F	63	O	9.2 - 17070 - 34K - 1.08%	16.95	9.3	31	22.4	1.93	57.7	64	160	75.00	1	2
P-078	F	70	O	9.2 - 60960 - 75K - 0.32%	43.01	8.2	29	15.5	1.28	35.6	181		47.01	1	2
P-082	M	42	O	9.3 - 16580 - 78K - 1.07%	6.21	7.2	23	17.0	1.35	43.0	152		60.00	0	2
P-098	M	56	O	8.3 - 10730- 23K - 1.26%	2.20	10.3	14	19.3	1.60	43.6	203		60.00	0	2
P-100	M	46	O	8.0 - 132140 - 65K - 0.68%	98.03	9.4	28	23.2	2.10	49.8	160	240	60.00	1	2
P-105	F	63	O	7.6 - 5,670 - 20K - 1.61%	1.37	8.7	53	14.7	1.18	36.4	344	171	39.35	0	1
P-525	M	69	O												
P-505	F	56	O												
P-520	M	67	O												
P-523	M	65	O												

ddimer.score	pt.score	fibrinogen.score	DIC.score	OvertDIC.01	Chemo.Rx	Ind.date	d7.WBC	d7.Hb	d7.PLT	d7.PT	d7.PT.INR	d7.APTT	d7.Fibrinogen	d7.FDP	d7.D-dimer	d7.plt.score
3	1	0	5	1	Ida+ATRA	2009-03-21	1.1	8.8	77	18.7	1.6	52.6	298	20	16.55	1
3	1	0	6	1	Ida+ATRA	2009-10-19	4.27	8.8	39	15	1.21	36.2	190			2
3	0	0	5	1	Ida+ATRA	2009-11-27	0.55	9.2	28	15.4	1.25	164				2
3	0	0	5	1	Ida+ATRA	2009-12-10	39.09	8.3	44	17.9	1.51	62	170			2
3	1	0	5	1	Ida+ATRA	2010-08-19	1.52	10.2	46	15	1.18	53.3				2
3	0	0	4	0	Ida+ATRA	2011-02-22	1.64	9.6	78	14.5	1.13	37.2	378		21.21	1
3	0	0	5	1	AIDA+ATRA	2011-11-25	0.38	9.3	62	14.1	1.08	35.5	219			1
3	0	0	5	1	Ida+ATRA	2010-06-16	0.73	9.2	35	15.2	1.2	37.7	157			2
3	0	1	5	1	AIDA+ATRA	2012-07-28	1.39	11.1	44	14.2	13.9	1.08	36.4		1.62	2
3	0	1	6	1	Ida+ATRA	2012-10-20	0.32	9.4	68	13.5	1.04	39	232		18.89	1
3	0	0	3	0	Ida+ATRA	2013-11-29	0.65	8.4	40	15.1	1.19	516				2
3	0	0	5	1	ATRA only?	2001-08-22	358.82	7.3	97	20.8	1.98	30.2				1
3	0	0	5	1	Ida+ATRA	2008-06-10	3.09	8.1	33	14.1	1.14	36.8				2
3	1	0	6	1	ATRA	2009-03-23	19.4	7.8	33	14.7	1.18	38.6	398	80	69.00	2
3	0	0	3	0	Ida+ATRA	2009-09-02	0.45	8	63	16.7	1.38	63.1	309	29.8		1
3	0	0	5	1	Ida+ATRA	2009-12-10	1.05	7.9	49	16.2	1.27	41.7	239			2
	0		0		Ida+ATRA	2010-08-14	6.71	9.2	10	17.3	1.43	55.9	361		12.96	2
	1	1	4		Ida+ATRA	2012-09-03	0.94	10.6	27	14.3	1.12					2
3	2	0	6	1	Ida+ATRA	2013-07-10	1.46	9.3	50	13.7	1.08	30.5	159			1
3	1	1	7	1	Ida+ATRA	2004-08-24	0.3	9.8	61	14.1	1.07	37.6	181			1
3	0	1	6	1	Ida+ATRA	2011-01-14	0.99	8.7	46	13	0.98	32.7	317		10.52	2
3	0	0	5	1	Ida+ATRA	2008-10-11	5.03	8.8	30	17.2	1.44	39.9	179			2
3	0	0	5	1	Ida+ATRA	2012-10-22	1.24	7.9	25	14.5	1.14	42.5	385	32.21	8.37	2
3	1	0	6	1	AIDA+ATRA	2012-10-24	1.96	13.3	106	16.7	1.37	32.3	153			0
3	0	0	5	1	AIDA+ATRA	2013-05-22	2.9	8.2	61	14.3	1.12	36.3	237			1
3	0	0	5	1	Ida+ATRA	2013-11-26	0.33	9.8	67	15.6	1.24	35.4	236	31.52	9.62	1
3	2	1	7	1	Ida+ATRA	2013-11-26	0.61	8	75	15.4	1.22	61.7	477		3.59	1
3	0	0	4	0	IA+ATRA	2013-11-29	0.26	9	47	15.7	1.25	48.1	293		1.63	2
3	0	0	5	1	IA+ATRA	2013-12-24	0.64	8.1	47	13.8	1.06	32.7	217		1.15	2
	1	0	3		IA+ATRA	2002-12-17	9.42	7.9	68	21.4	1.65	36.4	303			1
3	2	1	8	1	Ida+ATRA	2003-02-15	1.61	8.1	96	15.8	1.22	34.7	239			1
3	2	1	8	1	Ida+ATRA	2009-01-26	0.44	9.4	55	23.4	2.05	54	116	80		1
3	0	0	5	1	Ida+ATRA	2011-04-10	1.54	8.7	41	14.9	1.17	33.6	152			2
3	1	0	6	1	Ida+ATRA	2011-12-30	5.67	9	153	17.4	1.39	35				0
3	1	0	6	1	ATRA	2013-01-06	3.73	11.4	102	24.9	2.22	57.3	151		60.00	0
3	2	0	7	1	Ida+ATRA	2013-03-12	8.54	7.8	23	19.9	1.72	36.4	351	240	60.00	2
3	0	0	4	0	IA+ATRA	2013-08-03	0.03	9	28	17	1.43	42.5	528			2
					Ida+ATRA	2013-02-27										

d7.ddimer.score	d7.pt.score	d7.fibrinogen.score	d7.DIC.score	d7.OvertDIC.01	d28.WBC	d28.Hb	d28.PLT	d28.PT	d28.PT.INR	d28.APTT	d28.Fibrinogen	d28.FDP	d28.D-dimer	d28.plt.score	d28.ddimer.score
3	1	0	5	1	11.73	10	331	16.0	1.31	47.5				0	
	0	0	2		1.32	9	38	15.5	1.26	48.2	414			2	
	0		2		7.11	10	556	13.5	1.06	38.9	301			0	
	1	0	3		1.87	10	114	13.7	1.08	38.7	266		3	0	2
	0		2		2.83	10	80	13.4	1.01	43.8				1	
3	0	0	4	0	0.39	11	41	14.3	1.11	39.2	382		14	2	3
	0	0	1		0.96	10	105	14.7	1.12	40.8	445			0	
	0	0	2		2.45	10	133	15.3	1.22	41.6				0	
2	0	1	5	1	2.42	9	127	13.5	1.04	37.2				0	
3	0	0	4	0	0.61	9	52	14.4	1.10	44.7	406		6	1	3
	0		2		3.36	9	256	14.2	1.10	39.3	521			0	
	2		3												
	0		2		1.82	8	70	14.8	1.21	37.9				1	
3	0	0	5	1	0.88	9	48	18.7	1.60	50.5	255	20	18	2	3
	0	0	1		1.19	8	28	15.0	1.21	49.2	264			2	
	0	0	2		10.19	10	19	27.8	2.63	70.4	147		40	2	3
3	1	0	6	1	4.95	10	147	15.1	1.18	39.2				0	
	0		2		2.70	9	47	15.8	1.28	49.4				2	
	0	0	1		6.24	10	255	14.2	1.13	42.8	358			0	
	0	0	1		1.78	11	229	14.8	1.12	51.7	650			0	
3	0	0	5	1	0.73	10	84	14.4	1.12	36.8	426			1	
	1	0	3		2.75	10	232	14.6	1.18	33.4				0	
3	0	0	5	1	2.42	8	58	16.0	1.26	47.6	487			1	
	0	0	0		0.87	11	101	14.5	1.11	30.3	225			0	
	0	0	1		2.10	10	392	17.4	1.48	42.1	528	6	1	0	2
3	0	0	4	0	0.19	9	28	17.2	1.40	45.8				2	
2	0	0	3	0	5.68	9	77	14.9	1.17	49.6	290			1	
2	0	0	4	0	1.11	8	45	15.0	1.18	53.1	505			2	
2	0	0	4	0	2.17	11	332	14.0	1.08	33.6	228			0	
	2	0	3												
	0	0	1												
	2	0	3		23.08	11	42	23.1	2.01	62.1	234		17	2	3
	0	0	2		0.75	11	109	14.3	1.15	43.7	516			0	
	1		1												
3	2	0	5	1											
3	1	0	6	1											
	1	0	3		0.01	14	15	18.6	1.61	34.4	513		7	2	3

d28.pt.score	d28.fibrinogen.score	d28.DIC.score	d28.OvertDIC.01	Albumin	Creatinine	Uric Acid	Cluster	last.fu	rel.01	rel.01.date	exp.01	exp.date	Surv.m	Surv.d
0		0		3.1	4.08	9.7	MS1	2016-08-16	0		0	2016-08-16	#REF!	#REF!
0	0	2		4.7	0.59	1.3	MS1	2020-06-22	1	2011-02-28	0	2020-06-22	#REF!	#REF!
0	0	0		4.4	0.67	3.3	MS1	2017-08-10	0		0	2017-08-10	#REF!	#REF!
0	0	2	0	4.7	1.06	5.4	MS1	2019-01-18	0		0	2019-01-18	#REF!	#REF!
0		1		4.5	0.57	3.2	MS1	2016-05-24	0		0	2016-05-24	#REF!	#REF!
0	0	5	1	3.7	0.76	3.1	MS1	2019-10-17	0		0	2019-10-17	#REF!	#REF!
0	0	0		4.9	0.58	3.7	MS1	2018-07-31	0		0	2018-07-31	#REF!	#REF!
0		0		4	0.49	3.1	MS1	2020-03-12	0		0	2020-03-12	#REF!	#REF!
0		0		5.2	0.68	7.5	MS1	2018-01-09	0		0	2018-01-09	#REF!	#REF!
0	0	4	0	4.9	1.01	3.5	MS1	2019-08-08	0		0	2019-08-08	#REF!	#REF!
0	0	0		4.5	1.15	5.5	MS1	2019-09-19	0		0	2019-09-19	#REF!	#REF!
				3.7	0.6	2.9	MS1	2001-08-23	0		1	2001-08-23	#REF!	#REF!
0		1		3.8	0.83	3.5	MS1	2014-07-31	0		1	2014-07-31	#REF!	#REF!
1	0	6	1	2.8	0.96	3.2	MS1	2009-05-22	0		1	2009-05-22	#REF!	#REF!
0	0	2		3.9	5.01	7.2	MS1	2014-09-09	0		1	2014-09-09	#REF!	#REF!
2	0	7	1	3.7	0.73	5.4	MS1	2010-01-07	0		1	2010-01-07	#REF!	#REF!
0		0		2.1	0.61	5.4	MS1	2011-04-22	0		1	2011-04-22	#REF!	#REF!
0		2		4.2	0.79	5.1	MS1	2015-04-17	0		1	2015-04-17	#REF!	#REF!
0	0	0		4.4	1.21		MS1	2018-10-18	1	2018-01-15	1	2018-10-18	#REF!	#REF!
							MS1							
0	0	0		2.4	0.98	7.3	MS2	2011-09-15	0		0	2011-09-15	#REF!	#REF!
0	0	1		4.7	0.86	6.4	MS2	2020-08-17	0		0	2020-08-17	#REF!	#REF!
0		0		3.3	0.75	2.2	MS2	2020-08-10	0		0	2020-08-10	#REF!	#REF!
0	0	1		4.8	0.69	6.8	MS2	2018-08-20	0		0	2018-08-20	#REF!	#REF!
0	0	0		4.4	0.84	5.5	MS2	2020-08-06	0		0	2020-08-06	#REF!	#REF!
1	0	3	0	5.3	0.67	4.4	MS2	2020-07-08	0		0	2020-07-08	#REF!	#REF!
1		3		4.4	0.77	4.2	MS2	2020-08-19	0		0	2020-08-19	#REF!	#REF!
0	0	1		4.3	0.94	3.8	MS2	2019-10-10	0		0	2019-10-10	#REF!	#REF!
0	0	2		4.4	0.74	4.1	MS2	2019-07-30	0		0	2019-07-30	#REF!	#REF!
0	0	0		3.8	0.53	2.3	MS2	2020-04-14	0		0	2020-04-14	#REF!	#REF!
				4.9	0.9	5.7	MS2	2002-12-21	0		1	2002-12-21	#REF!	#REF!
				5	1.1	2.8	MS2	2003-02-21	0		1	2003-02-21	#REF!	#REF!
2	0	7	1	4	0.84	4.6	MS2	2009-02-12	0		1	2009-02-12	#REF!	#REF!
0	0	0		4	0.49	3.6	MS2	2013-08-28	1	2012-07-09	1	2013-08-28	#REF!	#REF!
				4.5	0.94	4.2	MS2	2012-01-08	0		1	2012-01-10	#REF!	#REF!
				4.7	1.33	4.2	MS2	2013-01-10	0		1	2013-01-10	#REF!	#REF!
				4.8	1.48	7.3	MS2	2013-03-21	0		1	2013-03-21	#REF!	#REF!
1	0	6	1	4.6	0.71	4.8	MS2	2013-08-13	0		1	2013-08-13	#REF!	#REF!
							MS2	2014-01-10	0		1	2014-01-10	#REF!	#REF!
							MS2							
							MS2							
							MS2							

Etc.
ARF on HD, under treatment R/O stress induced CMP
relapse, allo
r/o ATRA sydrom
TRMN, colon ca. s/p CTx.
Brain infraction, leukocytosis, R/O ATRA syndrome으로 사망
allo, Lung GVHD dead
Pneumonia, r/o Septic shock
DMCRF dead ,r/o ATRA toxicity (for CNS VS renal fuction), H/O severe tremor with decreased mentality
2005 HCC Dx. Therapy-related? ARF (renal failure), R/O fungal pneumonia
hematuria, melena, bleeding, dead
HAP, r/o vasculitis, **TRNM (t-AML on 2015-04-13;t-MDS on 2014-09-25)**
relapse, auto, septic shock
trustee
DIC
ICH, ICH, idarubicin stop, ATRA
SDH
HCC history
TRMN, breast ca s/p CTx
DIC, ICH
ICH
CTx. hold, ARDS, ARF, DIC, r/o ATRA syn, 2008 Cervical cancer TAH/BSO
Relapse (CNS & BM), septic shock
DIC, ICH, hopeless discharge
Septic shock, DIC, Indonesia travel (from 1 week prior to travel fever), Rhabdomyolysis
DIC, septic shock, ICH
DIC, s/p KT, ATRA syn, ARF (Resp.)
2007 HBV, HCC Dx. Liver failure dead, APL
trustee
trustee
trustee

Supplement Table4. mRNA sequencing statistics

Mean of				
Total read	Read length (bp)	Uniquely mapped reads number	Uniquely mapped reads (%)	Mapped length (bp)
34,525,658	200	28,186,972	88	199
